# Auxin and Cytokinin Metabolism and Root Morphological Modifications in *Arabidopsis thaliana* Seedlings Infected with *Cucumber mosaic virus* (CMV) or Exposed to Cadmium

**DOI:** 10.3390/ijms14046889

**Published:** 2013-03-26

**Authors:** Antonella Vitti, Maria Nuzzaci, Antonio Scopa, Giuseppe Tataranni, Tony Remans, Jaco Vangronsveld, Adriano Sofo

**Affiliations:** 1 School of Agricultural, Forestry, Food and Environmental Sciences, University of Basilicata, Viale dell’Ateneo Lucano 10, I-85100 Potenza, Italy; E-Mails: antonella.vitti@unibas.it (A.V.); maria.nuzzaci@unibas.it (M.N.); antonio.scopa@unibas.it (A.S.); giuseppe.tataranni@unibas.it (G.T.); 2 Environmental Biology Centre for Environmental Sciences, Hasselt University, Agoralaan, building D, B-3590 Diepenbeek, Belgium; E-Mails: tony.remans@uhasselt.be (T.R.); jaco.vangronsveld@uhasselt.be (J.V.)

**Keywords:** abiotic stresses, *Arabidopsis thaliana*, biotic stresses, cadmium, *Cucumber mosaic virus* (CMV)

## Abstract

*Arabidopsis thaliana* L. is a model plant but little information is available about morphological root changes as part of a phytohormonal common response against both biotic and abiotic stressors. For this purpose, two-week-old Arabidopsis seedlings were treated with 10 μM CdSO_4_ or infected with CMV. After 12 days the entire aerial parts and the root system were analyzed, and the presence of CMV or the accumulation of Cd were detected. Microscopic analysis revealed that both CMV and Cd influenced root morphology by a marked development in the length of root hairs and an intense root branching if compared to controls. Among the three treatments, Cd-treated seedlings showed a shorter root axis length and doubled their lateral root diameter, while the lateral roots of CMV-infected seedlings were the longest. The root growth patterns were accompanied by significant changes in the levels of indole-3-acetic acid, *trans*-zeatin riboside, dihydrozeatin riboside, as a probable consequence of the regulation of some genes involved in their biosynthesis/degradation. The opposite role on root development played by the phythormones studied is discussed in detail. The results obtained could provide insights into novel strategies for plant defense against pathogens and plant protection against pollutants.

## 1. Introduction

Many factors, both biotic (pathogens, insects, nematodes) and abiotic (e.g., wounds, pollutants, thermal, water and nutritional imbalances, environmental contaminants) are causes of plant stress and can decrease plant growth and productivity. Plants can react to these stressors through a series of constitutive and/or inductive mechanisms which result in the elimination or the limitation of the negative effects induced by the adverse factors.

Plant viruses are obligate parasites because require living tissue for their multiplication and spread, interfering with plant metabolism and/or competing for host plant resources. As a response to viral infection, plants may compensate with a broad range of cellular processes by up- or down-regulating certain genes, changing the levels of substances implicated in plant defense pathway, increasing the levels of reactive oxygen species (ROS), activating specific transcription factors, defense-regulated genes, heat-shock proteins, and enhancing the transport of macromolecules, enzymes, and phytohormones involved in defence signaling pathways (e.g., salicylic acid, SA; jasmonic acid, JA; ethylene, ET) [1–3]. On the other hand, the involvement of auxins (in particular of indole-3-acetic acid—IAA, the most widely auxin in plants) and of cytokinins (CKs) in viral pathogenesis has been poorly studied [4].

The ability of viruses to significantly interfere with endogenous hormone levels is closely related to a range of symptoms caused by an abnormal growth, as stunting, galls, enations, tissue distortions [5]. Particularly, *Cucumber mosaic virus* (CMV) is the plant virus with the largest host range of all RNA viruses, therefore its spreading on crop plants may cause serious economic damages. It has been widely studied because it represents an interesting model from a physico-chemical point of view, as it causes a wide range of symptoms, especially yellow mottling, distortion and plant stunting [6,7].

Cadmium is a major environmental contaminant that enters human food via accumulation in crop plants and is considered as being one of the most ecotoxic metals that exhibits adverse effects on a wide range of biological processes in humans, animals, and plants [8]. Cadmium levels in soils are generally low (an average of 0.3 μM) and, for this reason, plants usually are not exposed to toxic levels of this metal under normal environmental conditions [8]. Cadmium, even at low concentrations (<1 μM in the soil solution), is efficiently absorbed by plant roots, translocated via xylem, and compartmentalized in vacuoles, and it influences the transcription of several genes [8,9]. Cadmium levels of 1–5 μM in the soil solution are sufficient to retard root growth, whereas contaminated soils contain mean Cd concentrations of 10 μM [8,9].

Interestingly, *Arabidopsis thaliana* plants exposed to Cd often resemble, with their peculiar growth pattern, plants altered in phytohormone metabolism [10,11]. In the response to Cd, a key role is played by abscisic acid (ABA) [12], JA [13] and ET [14], but other plant growth regulators (e.g., auxins and cytokinins) could be involved, as demonstrated by other authors [15,16]. Considerable effort has also been directed at clarifying the processes and factors contributing to IAA and CK homeostasis during Cd exposure, but the entire picture remains to be elucidated, as the synthesis of these hormones is regulated in response to different and complex signaling pathways [17]. Moreover, with the exception of some studies [16,18,19], the action of IAA and CKs on root morphology and architecture in Arabidopsis has not been deeply examined.

The aim of this work was to investigate the roles of IAA and CKs in Arabidopsis seedlings exposed to Cd or infected by CMV. We hypothesized that changes of the metabolism of IAA and CKs in Arabidopsis could be part of a common response to both biotic (CMV) and abiotic (Cd) stresses, and that the changes in their levels could be one of the causes of the morphological modifications observed in plants subjected to these two stressors.

## 2. Results and Discussion

**Figure 1:**
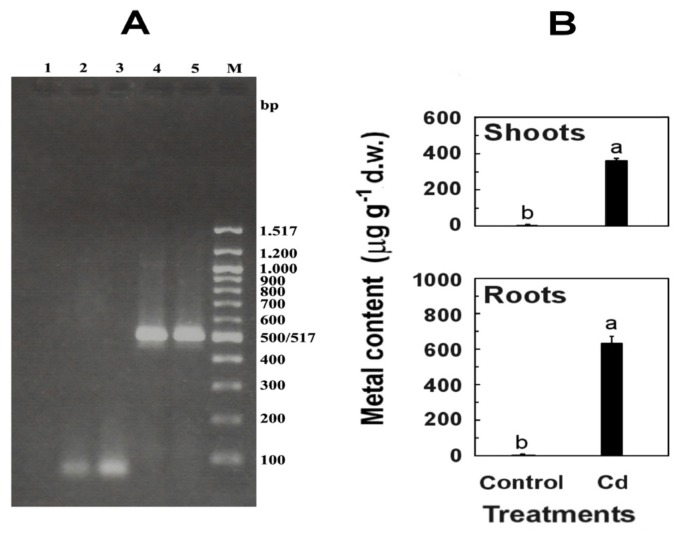
(**A**) Detection of *Cucumber mosaic virus* (CMV)-Fny in shoots and roots of Arabidopsis by Reverse Transcription Polymerase Chain Reaction (RT-PCR). 1, no fragment derived from negative control; 2, no fragment derived from shoots of healthy control; 3, no fragment derived from roots of healthy control; 4, DNA fragment of 513 bp derived from shoots of CMV-Fny infected *Arabidopsis* seedlings; 5, DNA fragment of 513 bp derived from roots tissues of CMV-Fny infected Arabidopsis seedlings; M, 100 bp DNA Ladder (New England BioLabs Inc., USA); (**B**) Levels of Cd in shoots (above) and roots (below) of Arabidopsis seedlings treated with 10 μM CdSO_4_ (Cd). Mean values (*n* = 10) ± SE with different letters are significantly different at *p* ≤ 0.05, according to one-way analysis of variance (ANOVA) with Fisher’s LSD test.

RT-PCR analysis of roots and shoots infected systemically with CMV-Fny, collected 12 days after inoculation, showed the expression of RdRp gene (513 bp) (Figure 1), thus confirming the ability of virus to multiply and spread throughout the plant.

Cadmium-exposed seedlings efficiently absorbed Cd, that accumulated in roots and was partly transported and stored in shoots, where it was found in lower concentration (57% of the value found in roots) (Figure 1). This high percentage is due to the fact that, at Cd levels up to 50 μM, the rhizodermic Cd transport through the symplast and the apoplastic route of Cd through the cortex is efficient if compared to other related species [20].

**Figure 2:**
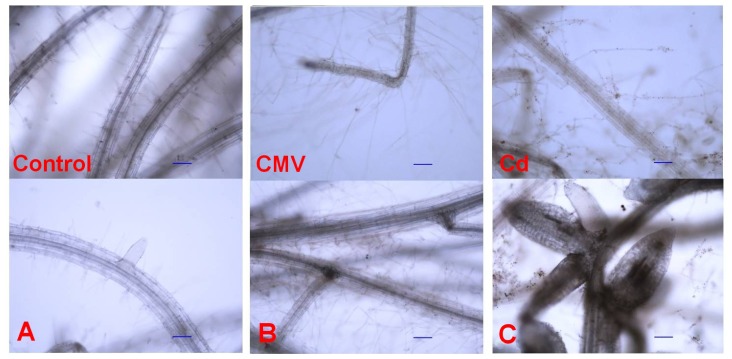
Root morphology (above: root hairs; below: branching) of *Arabidopsis* seedlings untreated (**A**; Control), infected with CMV (**B**; CMV), and treated with 10 μM CdSO_4_ (**C**; Cd). Roots were observed at 100× magnification; scale bars = 0.01 cm.

Generally, the presence of Cd inhibits root elongation and influences root anatomy, but apoplastic movement of Cd to the xylem can be restricted by the development of the exodermis, endodermis, and other extracellular barriers [21]. On the other hand, following a viral infection and the accumulation of viral proteins host cells undergo defects in root growth and development [6]. Our microscopy observations pointed out that root morphology was strongly affected by both CMV infection or Cd exposure (Figure 2B,C).

Quantitative parameters were measured to highlight the differences observed microscopically among the treatments (Table 1). CMV and Cd induced a marked increase in the length of root hairs, 3–4 times more than the controls (Table 1 and Figure 2). Furthermore, the whole root system of Cd-treated seedlings resulted less expanded. Indeed, its main axis length was 10 cm, compared to 16 and 13 cm of CMV-infected and control seedlings, respectively (Table 1 and Figure 2). There were no changes in main root diameter among the three treatments (Table 1 and Figure 2). Both CMV and Cd brought about significantly greater root branching if compared to controls, as the mean distance between adjacent root branches declined by 36% after CMV infection and by 17% under Cd exposure, compared to the mean control value of 0.35 cm (Table 1 and Figure 2). Clusters of 3–5 new lateral roots, emerging from the same point, were particularly evident in Cd-treated seedlings (Figure 2C). The lateral roots of the seedlings exposed to Cd showed characteristic traits, as they were short and fleshy, and a diameter that was twice that of both CMV-infected and control seedlings (Table 1 and Figure 2). The lateral roots of CMV-infected seedlings were the longest (0.458 cm), if compared to the values of 0.038 and 0.017 cm observed in Cd-treated and control seedlings, respectively.

**Figure 3:**
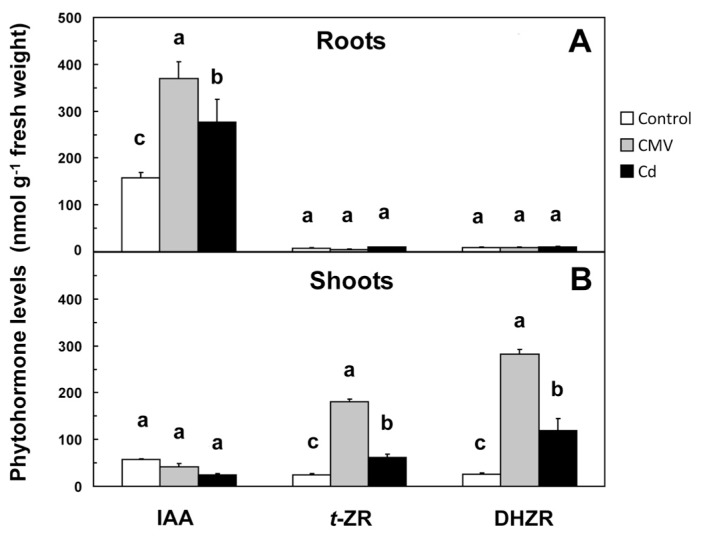
Levels of indole-3-acetic acid (IAA), *trans*-zeatin riboside (*t*-ZR) and dihydrozeatin riboside (DHZR) in roots (**A**; above) and shoots (**B**; below) of Arabidopsis seedlings infected with CMV (CMV; grey columns), treated with 10 μM CdSO_4_ (Cd; black columns), and untreated (Control). Mean values (*n* = 10) ± SE with different letters are significantly different between treatments at *p* ≤ 0.05, according to one-way ANOVA with Fisher’s LSD test.

The various root growth patterns observed after CMV infection or Cd application were accompanied by significant changes in the root levels of IAA, *t*-ZR and DHZR (Figure 3A) as a probable consequence of the regulation of some genes involved in their production/degradation (Table 2).

It is known that the activation of defenses induced by abiotic stresses or pathogens in plants is mediated through the synthesis of molecules with signal functions, including plant growth regulators. In particular, indole-3-acetic acid (IAA) is the most widely auxin in plants [11,17,22]. Key components of the cell cycle and signal-transduction pathways that modulate auxin-dependent lateral roots initiation and elongation have been identified in Arabidopsis [23].

Abdala *et al.*[24] were the first to detect the presence of high IAA levels in diseased tissues and in not yet well developed enations inducted by *Mal de Rio Cuarto virus* (MRCU) in maize. More recently, a study about *Tobacco mosaic virus* (TMV)-induced disease symptoms on tomato and Arabidopsis attributed to the ability of the viral replicase protein to disrupt the localization and function of interacting transcriptional repressors called Auxin/Indole-3-acetic acid (Aux/IAA) proteins in order to reprogram the cellular environment of older cells and make it more suitable for virus replication and spread [25]. In another investigation, the hormonal status of potato plants infected with *Potato virus Y* (PVY) was analyzed, and it was demonstrated that IAA was not implicated in early responses of plant against the virus [26]. Our results show that CMV determined an increase in IAA levels in roots from 157.29 to 370.33 nM g^−1^ of fresh weight (f.w.) (Figure 3A), accompanied by a significant up-regulation (4.3-fold) of the transcription of the gene encoding nitrilase (*AtNIT*) (Table 2), the enzyme catalyzing the final regulatory reaction of IAA biosynthetic pattern having indole-3-acetonitrile as substrate. In the roots of CMV-infected seedlings, the expression of *AtAAO* gene, encoding for the enzyme aldehyde oxidase, catalyzing the oxidation of indole-3-acetaldehyde to IAA, did not change significantly compared to controls (Table 2). These results suggest that the CMV-induced enhancement of *AtNIT* expression is one of the possible reasons for the increase of IAA concentration. At level of shoots of CMV-infected seedlings, *AtNIT* did not show significant changes if compared to controls, whereas *AtAAO* was significantly up-regulated (3.1-fold) (Table 2). Furthermore, the absence of significant differences in shoot IAA levels between controls and CMV-infected seedlings occurred (Figure 3B).

Actually, there are few studies on the relationships between Cd, or other heavy metals, and IAA in Arabidopsis or in related species [10,16]. For example, Pasternak *et al.*[10] observed that in Arabidopsis metal-exposed plants root hair density was significantly increased and an acceleration of the emergence of lateral roots occurred, concluding that phenotypes of Arabidopsis seedlings exposed to CuSO_4_ resemble those of plants altered in auxin metabolism. In this study, Cd application determined a significant 76% increase in IAA levels in roots if compared to controls (Figure 3A), likely due to the up-regulation (3.4-fold) of *AtNIT* (Table 2). In contrast, the expression of the same gene in shoots was not significantly affected (Table 2). Moreover, Cd did not determine changes in *AtAAO* expression either in shoots or in roots if compared to controls (Table 2). This suggests that the observed changes of IAA levels in roots can be mainly due to *AtNIT* up-regulation.

*Trans*-zeatin riboside (*t*-ZR) and dihydrozeatin riboside (DHZR), two of the most important natural cytokinins (CKs), are implied in inhibiting xylem formation and root growth, releasing inactive lateral buds from growth inhibition, promoting leaf expansion, and delaying senescence [27]. In the presence of auxins, CKs are able to promote cell division in plants, and exogenous cytokinin applications induce cell division in tissue cultures [28]. Sziraki *et al.*[29] were the first to report that the systemic infection by CMV caused an increase in the CK content in leaves of tobacco. More recently, Clarke *et al.*[30] demonstrated that the supplementation of CKs in the xylem stream was able to inhibit the replication of a potexvirus in bean. In Arabidopsis, the gene encoding isopentenyltransferases (*AtIPT*), catalyzing the isopentenylation of AMP, is of basic importance in the regulation of CKs biosynthesis, whereas the gene encoding for the main enzyme for CKs degradation pathway is cytokinin oxidase (*AtCKX*) [31].

In this study, the roots of CMV-infected seedlings showed an up-regulation of *AtCKX* (21.5-fold) and a down-regulation of *AtIPT* (0.4-fold), whereas the opposite was observed in shoots (0.1-fold for *AtCKX* and 45.3-fold for *AtIPT*) (Table 2). After Cd treatment, the transcription of *AtIPT* was strongly up-regulated in shoots (13.5-fold) and did not change in roots, whereas *AtCKX* was up-regulated in roots (7.6-fold) and down-regulated in shoots (0.1-fold) (Table 2). The observed trends of the transcription of the genes involved in CKs metabolism in CMV-infected and Cd-treated seedlings suggest that CKs turnover in shoots and roots was mainly regulated by the expression of both *AtIPT* and *AtCKX*, respectively. Particularly, in the shoots of seedlings grown in the presence of either biotic (CMV) or abiotic (Cd) stressors, the increase in *AtIPT* expression accompanied by a low *AtCKX*, expression level is consistent with the proportional increase of *t*-ZR and DHZR in those tissues (Table 2 and Figure 3B). On the other hand, CKs levels in roots of CMV-infected and Cd-treated seedlings resulted to be not significantly different from those found in the controls (Figure 3A). Under normal conditions, CKs synthesis occurs in the root cap cells and then these hormones are transported up to shoot organs. In our case, plants were likely able to keep the concentration of CKs in the shoot within physiological limits, at least partly, by the up-regulation of *AtIPT* under conditions of insufficient CKs supply from the root, according to Miyawaki *et al.*[32]. Our findings not only support those statements, but they also suggest a possible common response in *Arabidopsis* plants regarding CKs homeostasis under pressure of both biotic (CMV) and abiotic (Cd) stresses.

According to Aloni *et al*. [33], the up-regulation of *CKX* gene in the roots, that is what occurred in our study, results in an enlarged root meristem, formation of lateral roots closer to the root apical meristem, increased root branching and promotion of adventitious root formation.

## 3. Experimental Section

### 3.1. Plant Material and Experimental Design

Seeds of *Arabidopsis thaliana* L. (Columbia ecotype; Col-0) were sterilized using 50% (*v*/*v*) ethanol for 5 min followed by 5 min of 1% Na-hypochlorite and finally rinsed with sterile dH_2_0 before imbibition on moist filter paper at 4 °C for 24 h in the dark. Then, seeds were put in polyethylene containers (36 per container) filled with sterilized sand (mean particle size = 0.25 mm), and frequently moistened with 300 mL of one-quarter strength Hoagland liquid medium (2.53 mM KNO_3_, 0.75 mM Ca(NO_3_)_2_·4H_2_O, 0.50 mM NH_4_H_2_PO_4_, 0.50 mM MgSO_4_·7H_2_O, 4.10 mM FeSO_4_·7H_2_O, 2.03 mM Na_2_-EDTA, 11.58 mM H_3_BO_3_, 2.28 mM MnCl_2_·4H_2_O, 0.08 mM CuSO_4_·5H_2_O, 0.15 mM H_2_MoO_4_·H_2_O, 0.40 mM ZnSO_4_·7H_2_O). The Hoagland solution was continuously replaced in order to maintain a constant volume of 300 mL and to keep the roots moistened. Throughout the experiment, the pH of the Hoagland solution was constantly measured and no significant changes were observed (mean value of 6.5). Two-week-old seedlings were treated with 10 μM CdSO_4_ (the concentration of Cd used simulated that usually present in the soils contaminated by this metal [8]) or mechanically inoculated with CMV-Fny, purified as described by Lot *et al.*[34]. After 12 days of exposure to heavy metal or after the infection, the entire aerial parts and the root system were separated and immediately used for the following analyses. The period of 12 days was chosen on the basis of the acute phase of CMV infection (maximum symptoms expression) observed, and for avoiding toxic Cd accumulation in plants. Healthy seedlings were used as control.

### 3.2. Morphological Analysis

The fresh root systems, kept in the Hoagland solution to avoid drying, were mounted on slides and observed at different magnifications using a compound optical microscope (Eclipse 80i; Nikon, Tokyo, Japan) under transmitted light, and then photographed (Digital Camera DS-Fi1 equipped with NIS-Elements Imaging Software; version 3.0; Nikon: Tokyo, Japan, 2010). Images were analyzed to compare root morphology and evaluate descriptive parameters. Descriptive and quantitative parameters were used to underline differences observed microscopically among the treatments. The number of root hairs per Square centimeter of root surface was counted and hair length was measured. Main and lateral root mean diameter at 0.02 cm from root tip, lateral root length and root branching, this latter defined as the mean distance between two adjacent root branches, were also measured.

### 3.3. Cadmium Determination

Roots were previously and carefully cleaned twice with 10 mM CaCl_2_ and then twice with distilled water, according to Lasat *et al.*[35]. Explants of roots and shoots (1 g) were digested in a HNO_3_:H_2_O_2_ solution (5:1 *v*/*v*) using a high performance microwave digestion unit (MLS-1200 Mega, Milestone Inc., Shelton, CT, USA). The levels of Cd were determined by inductively coupled plasma-atomic emission spectrometry (ICP-OES; model iCAP 6000, Thermo-Scientific, Cambridge, UK). Blanks (only HNO_3_ and H_2_O_2_) and a standard stock solution of 50 ppm was analyzed for reference purposes. Results were expressed on a dry weight tissue basis (ppm).

### 3.4. Auxin and Cytokinin Extraction and Determination

An explant (1 g) of shoot or root tissue was ground into powder with liquid nitrogen with a mortar and pestle, and put in a tube. To each tube, 2.5 mL extraction solvent (2-propanol/H_2_O/HCl 37%; 2:1:0.002, *v*/*v*/*v*) was added. The tubes were shaken at a speed of 100 rpm for 30 min at 4 °C. To each tube, 2.5 mL of dichloromethane was added, and then the samples were shaken for 30 min at 4 °C and centrifuged at 13,000× *g* for 5 min. After centrifugation, two phases were formed, with plant debris between the two layers, so 1.0 mL of the solvent from the lower phase was transferred using a Pasteur pipette into a screw-cap vial, and the solvent mixture was concentrated using an evaporator with nitrogen flow. Finally, the samples were re-dissolved in 0.1 mL methanol and stored at −20 °C before quantitative analysis.

The quantitative determinations of IAA, and *trans*-zeatin riboside (*t*-ZR) and dihydrozeatin riboside (DHZR) were carried out by high performance liquid chromatography coupled with mass spectrometry (Shimadzu LCMS-2020 equipped with an ESI source, with two LC-2020AD pumps, CBM-20A controller and SIL-20A MS-2020 auto-sampler; Shimadzu Co., Kyoto, Japan). The chromatographic separation was conducted using a Shim-Pak XR-ODS column, 2 mm × 50 mm (Shimadzu) and a mobile phase of 0.1% formic acid in water (Solvent A) and 0.1% formic acid in methanol (Solvent B) delivered in gradient elution mode at a flow rate of 0.3 mL min^−1^. The elution program used was as follows: 0–1 min, 30% B; 1–5 min, 80% B; 5–10 min, 100% B; 10–15 min, 30% B. Mass scans were measured from *m*/*z* 150 up to *m*/*z* 400, at 350 °C interface temperature, 230 °C DL temperature, ±4500 V interface voltage, neutral DL/Qarray, using N_2_ as nebulizing gas. Spectra of IAA, *t*-ZR and DHZR were acquired in the positive ionization mode. Pure standards of each phytohormone (Duchefa Biochemie B.V., Haarlem, The Netherlands) were used for identification analysis. The amounts of plant phytohormones in the samples were determined by calculating the correction factor of each detected plant hormone in comparison with its corresponding internal standard. The internal standards used were the following (OlChemIm Ltd., Olomouc, Czech Republic): [^2^H_5_] indole-3-acetic acid (cat. no. 0311533); [^2^H_5_] *trans*-zeatin riboside (cat. no. 0300313); [^2^H_3_] dihydrozeatin riboside (cat. no. 0300613).

### 3.5. Total RNA Extraction

Total RNA from Arabidopsis shoot and roots was extracted by TRIzol^®^ Reagent (Invitrogen, Milan, Italy). Tissues (100 mg) deriving from control, CMV-infected and Cd-treated seedlings were ground with mortar and pestle in 1 mL of sterile RNase-free water. To 300 μL of crude extract, 1 mL of TRIzol^®^ Reagent was added. The sample was homogenized and the following procedure for the dissociation of nucleoprotein complexes, phase separation, RNA precipitation, RNA washing and RNA re-dissolution were carried out as described by the manufacturer.

### 3.6. Reverse Transcription Polymerase Chain Reaction (RT-PCR) Analysis

In order to assess the presence of CMV in the plant tissues, an amount of 500 ng of total RNA extracted from both roots and shoots of CMV-infected *Arabidopsis* seedlings was reverse-transcribed and amplified in a single tube using the SuperScript^™^ III One-Step RT-PCR System with Platinum^®^*Taq* DNA Polymerase (Invitrogen, Milan, Italy). The RT-PCR reaction mixture (final volume of 50 μL) was prepared according to Vitti *et al.*[36], using an annealing temperature of 55 °C. The following couples of primers were used: P_RevRep_ (5′-TAACCTCCCAGTTCTCACCGT-3′), complementary to position 1895–1915 of the CMV-Fny RNA-dependent RNA polymerase (RdRp) gene, and P_ForRep_ (5′-CCATCACCTTAGCTTCCATGT-3′), homologous to the position 1403–1423 of the CMV-Fny RdRp gene, according to Grieco *et al.*[37]. The PCR fragments were fractionated on a 1.2% agarose gel and stained with SYBR Safe^™^ DNA gel stain (Invitrogen).

### 3.7. Real-Time Reverse Transcription PCR (qRT-PCR)

Priming of the cDNA reaction from the RNA template was carried out starting from an amount of 1 μg of total RNA extracted. The reaction was performed using the First-Strand cDNA Synthesis Kit (GE Healthcare, Chalfont St Giles, Buckinghamshire, UK), in a final volume of 15 μL, by using the random hexadeoxynucleotides pd(N)_6_, according to the manufacturer’s instructions. Primer pairs were designed for the real-time PCR amplification of sequences belonging to the genes known for having a key regulatory role in the biochemical pathways of IAA and CKs [31,33,38] (Table 3).

Real-Time PCR was performed in an optical 96-well plate with a 7500 Fast sequence detection system (Applied Biosystems by Life Technologies, Monza, Italy) using “fast mode” universal cycling conditions, followed by the generation of a dissociation curve to check for specificity of amplification. Reactions contained Fast SYBR Green Master Mix (Applied Biosystems), 300 nM of a gene specific forward and reverse primer (Table 3) and 2 μL of the diluted cDNA in each reaction.

Relative expression for genes of interest in each sample was calculated as 2^−ΔCq^, and normalized by the geometric average of 2^−ΔCq^ values for three reference genes per sample. Reference genes At5g15710 (F-box protein), At2g28390 (SAND family protein), and At5g08290 (mitosis protein YLS8) for normalizing gene expression data when plants are exposed to excess metals were identified previously [9].

### 3.8. Statistical Analysis

Statistical analysis was performed by analysis of variance (ANOVA) with SAS software (SAS Institute, Cary, NC, USA). Significant differences for gene expression analysis were determined at *p* < 0.05, according to Tukey-Kramer post-test after Shapiro-Wilk normality test. Significant differences for the remaining analyses were determined at *p* ≤ 0.05, according to Fisher’s LSD test. The number of measured samples is specified throughout the text, and in table and figure captions.

## 4. Conclusions

It is known that root growth and rooting are stimulated by IAA and inhibited by low concentrations of CKs. In this experiment, both CMV and Cd determined increased in auxin/cytokinins ratios in roots (IAA/(*t*-ZR + DHZR) = 9.8, 30.0 and 14.2 in controls, Cd-treated and CMV-infected seedlings, respectively). This hormonal ratio closely regulates lateral roots growth controlling the emergence of root primordia, so explaining the well developed root system of Cd-treated and CMV-infected seedlings (Table 1).

The opposite role played by IAA and CKs in root development can give an explanation of the observed change in root morphology in the three treatments. In metal-treated seedlings, Cd-related increase in root IAA (Figure 3A), likely due to *AtNIT* up-regulation in roots (Table 2), promoted lateral root formation and growth, so increasing root branching (Table 1). As pointed out by Schützendübel *et al*. and Dŭrčeková *et al*. [18,19], it is probable that Cd inhibited main root growth as a consequence of Cd-stimulated premature root differentiation and development, involving xylogenesis, induction of lateral roots, and growth of lateral roots and root hairs. The increase in lateral root diameter (Table 1 and Figure 2C) could be a consequence of Cd-induced xylogenesis, that act as a barrier to protect root from Cd, while the increasing of root branching could be considered as an “escaping” strategy of the roots in order to find areas free of Cd.

The response of Arabidopsis roots to CMV was very similar to that observed for Cd, as a high proliferation of long lateral roots and a rise in root hair length were observed (Table 1), even if not accompanied by an increase in lateral root diameter. The CMV-induced alterations in hormonal balance (Figure 3) and, as a consequence, in root morphology (Figure 2B and Table 1) could be an adaptative response induced by CMV. In this way, CMV could benefit from a greater root surface area, that in turn results in increased absorptive surface by branched roots and enhanced water and nutrient uptake capacity of the host plant. From the analysis of our results, it appeared that morphological root changes could be part of a hormonal common response of Arabidopsis plants against both biotic (CMV) and abiotic (Cd) stressors.

It is known that one of the main effects of the oxidative stress is the accumulation of reactive oxygen species (ROS). In particular, a wide range of both abiotic and biotic stresses results in H_2_O_2_ production [39]. Very recently, it was demonstrated that the electrochemical quantitative determination of extracellular H_2_O_2_ represents a real-time marker of oxidative stress [40]. The study of ROS levels to obtain more information about biological functions in Arabidopsis planned in our next experiments, together with the morphological modifications of root growth here detected, could provide insights into novel strategies for plant defense against pathogens and plant protection against pollutants. Indeed, it should be possible to consider the use of Arabidopsis also as morphological and biochemical bio-indicator for monitoring plant stress conditions reflecting environmental modifications.

## Figures and Tables

**Table 1:** Root growth parameters of Arabidopsis seedlings infected with CMV (CMV), treated with 10 μM CdSO_4_ (Cd), and untreated (Control).

	Main root axis length (cm)	Main root diameter (cm)	Root hairs (number cm^−2^)	Root hair length (cm)	Lateral root length (cm)	Lateral root diameter (cm)	Distance between root branches (cm)
Control	13 ± 3^a^	0.013 ± 0.002^a^	19172 ± 11969^a^	0.017 ± 0.005^b^	0.017 ± 0.004^c^	0.008 ± 0.001^b^	0.347 ± 0.077^a^
CMV	16 ± 3^a^	0.013 ± 0.006^a^	27677 ± 19367^a^	0.066 ± 0.039^a^	0.458 ± 0.130^a^	0.009 ± 0.001^b^	0.126 ± 0.008^b^
Cd	10 ± 2^b^	0.012 ± 0.004^a^	15362 ± 9186^a^	0.052 ± 0.013^a^	0.038 ± 0.006^b^	0.023 ± 0.002^a^	0.058 ± 0.008^c^

Mean values (*n* = 10) ± SE in the same column followed by different letters are significantly different at *p* ≤ 0.05, according to one-way ANOVA with Fisher’s LSD test.

**Table 2:** Expression levels of the genes involved in auxin and cytokinin metabolism measured by qRT-PCR (controls = 1.00) in roots and shoots of Arabidopsis seedlings infected with CMV (CMV), treated with 10 μM CdSO4 (Cd), and untreated (Control).

		*AtNIT*	*AtAAO*	*AtIPT*	*AtCKX*
Roots	Control	1.00 ± 0.29^b^	1.00 ± 0.05^a^	1.00 ± 0.23^a^	1.00 ± 0.39^a^
	CMV	4.32 ± 0.34^a^	0.78 ± 0.16^a^	0.36 ± 0.03^a^	21.54 ± 12.24^a^
	Cd	3.40 ± 0.96^a^,^b^	0.74 ± 0.06^a^	0.71 ± 0.21^a^	7.64 ± 3.84^a^
Shoots	Control	1.00 ± 0.25^a^	1.00 ± 0.04^b^	1.00 ± 0.05^a^	1.00 ± 0.01^a^
	CMV	3.10 ± 2.04^a^	3.00 ± 0.15^a^	45.32 ± 24.52^a^	0.12 ± 0.08^b^
	Cd	1.82 ± 1.03^a^	1.73 ± 0.25^b^	13.46 ± 4.92^a^	0.12 ± 0.01^b^

Mean values (*n* = 3) ± SE in the same column followed by different letters are significantly different at *p* ≤ 0.05, according to one-way ANOVA with with Tukey-Kramer post-test.

**Table 3:** Primers used for the amplification of the gene sequences involved in auxin and cytokinin metabolism.

Gene name	Accession number (TAIR Database)	Primer type	Primer sequence (5′→3′)
*Arabidopsis thaliana* nitrilase (*AtNIT*)	AT3G44310	Forward	TTGCTGGGAGAATAGGATGC
		Reverse	TTGCCATTCTTTCGAACCAT

*Arabidopsis thaliana* aldehyde oxidase (*AtAAO*)	AT5G20960	Forward	GGAGGAAGACATCCGATGAA
		Reverse	CGGTTAACCCTGCATCAAGT

*Arabidopsis thaliana* isopentenyltransferase (*AtIPT*)	AT3G23630	Forward	GCCGGTGGATCAAACTCTTA
		Reverse	AACGTCGACCCAAATGAAAC

*Arabidopsis thaliana* cytokinin oxidase (*AtCKX*)	AT2G41510	Forward	GACCACCAATTCCACCATTC
		Reverse	TTAAGAAGACGGCGGAGAAA

## Abbreviations

*AAO*: aldehyde oxidase
*CKX*: cytokinin oxidase
DHZR: dihydrozeatin riboside
IAA: indole-3-acetic acid
*IPT*: isopentenyltransferase
*NIT*: nitrilase
*t*-ZR: *trans*-zeatin riboside
